# A Cross-Sectional Study of Colonization Rates with Methicillin-Resistant *Staphylococcus aureus* (MRSA) and Extended-Spectrum Beta-Lactamase (ESBL) and Carbapenemase-Producing *Enterobacteriaceae* in Four Swiss Refugee Centres

**DOI:** 10.1371/journal.pone.0170251

**Published:** 2017-01-13

**Authors:** Rein Jan Piso, Roman Käch, Roxana Pop, Daniela Zillig, Urs Schibli, Stefano Bassetti, Dominik Meinel, Adrian Egli

**Affiliations:** 1 Medical Clinic, Cantonal Hospital of Olten, Olten, Switzerland; 2 Bakt Institut Olten BIO AG, Olten, Switzerland; 3 Division of Internal Medicine, University Hospital Basel, Basel, Switzerland; 4 Clinical Microbiology, University Hospital Basel, Basel, Switzerland; 5 Applied Microbiology Research, Department Biomedicine, University of Basel, Basel, Switzerland; Chang Gung Memorial Hospital, TAIWAN

## Abstract

**Background:**

The recent crisis of refugees seeking asylum in European countries challenges public health on many levels. Most refugees currently arrive from Syria, Afghanistan, or Eritrea. Data about multidrug resistant bacteria (MDR) prevalence are not present for these countries. However, when entering the European heath care systems, data about colonisation rates regarding highly resistant bacterial pathogens are important.

**Methods:**

We performed a cross-sectional screening in four Swiss refugee centres to determine the colonization rates for MRSA and ESBL- and carbapenemase-producing *Enterobacteriaceae*. We used pharyngeal, nasal, and inguinal swabs for MRSA and rectal swabs and urine for ESBL and carbapenemase screening using standard microbiological procedures. Whole genome sequencing (WGS) was used to determine the relatedness of MRSA isolates with high resolution due to a suspected outbreak.

**Results:**

41/261(15.7%) refugees were colonized with MRSA. No differences regarding the country of origin were observed. However, in a single centre significantly more were colonized, which was confirmed to be a recent local outbreak. 57/241 (23.7%) refugees were colonized with ESBL with significantly higher colonisation in persons originating from the Middle East (35.1%, p<0.001). No carbapenemase producers were detected.

**Conclusion:**

The colonisation rate of the refugees was about 10 times higher for MRSA and 2–5 times higher for ESBL compared to the Swiss population. Contact precaution is warranted for these persons if they enter medical care. In cases of infections, MRSA and ESBL-producing *Enterobacteriaceae* should be considered regarding antibiotic treatment choices.

## Introduction

As poverty, violent conflicts, or persecution of minorities remain important problems throughout the world, migration to countries with stable political situation or higher income will hardly diminish. According to the United Nations Refugee Agency (UNHCR), about 42’500 persons are forced to leave their homes every day due to conflict and persecutions [1]. While the majority of the worldwide 60 million refugees are displaced within the country of origin or distributed in neighbouring countries, developed countries provide asylum to 25% of the global total [1].

Although the data on prevalence of highly resistant bacteria in the origin countries of the refugees is not known, a worldwide increase of antibiotic resistance is documented [2]. Important gaps in surveillance exist and therefore, the colonization status with multi-drug resistant (MDR) bacteria of asylum seekers coming to developed countries remains largely unknown. The travel conditions promote the possibility of transmission from one person to another e.g. by overloaded boats without sanitary facilities, crowded refugee camps with poor hygiene conditions, and passage through countries with high rates of MDR bacteria such as Greece and Italy [3–5]. Screening for methicillin-resistant *Staphylococcus aureus* (MRSA) and extended-spectrum beta-lactamase (ESBL)-positive *Enterobacteriaceae* in refugees is controversially discussed as screening is not feasible for all refugees and the costs are high [6].

Many refugees seek medical attention in Western countries. Colonisation or infection with MDR bacteria may impact the therapeutic and infection control management. In addition, MDR bacteria possess a potential public health threat. In the current situation, more detailed data is certainly needed. In Switzerland, refugee centres are rather small (40 to 150 persons) and widely spread over the country. Once in Switzerland, refugees will be placed in one of the seven national arrival centres, where a nurse performs the initial health assessment without further detailed clinical, radiological, or laboratory investigations. Refugees stay in the arrival centres for one to three weeks and are then transferred in smaller cantonal centres. These centres often have collaborations with a physician practitioner or local hospital, but no centralized and standardized “migrant health” care is provided. The rapid transfer from primary to follow-up centres often causes difficulties in transferring previously collected medical information and thus causes an important gap especially in the context of transmittable infectious diseases such as MDR bacteria. Due to this unclear situation, some hospitals use contact precautions and procedures for patients from a refugee centre.

We assessed the cross-sectional rates of colonization of MRSA and ESBL as well as carbapenemase producing *Enterobacteriaceae* in four cantonal refugee centres in North-western Switzerland. We explored weather the colonization rate is linked to the refugee`s region of origin and other risk factors, such as superficial wounds. We used whole genome sequencing (WGS) to assess the relatedness of isolates and provided evidence for a suspected MRSA-transmission event in one refugee centre. In addition, we determined the rate of Panton-Valentine leukocidin (PVL)-positive MRSA strains from WGS data, as this toxin is a major virulence factor for pathogen transmission and more severe clinical infections [7].

## Patients and Methods

### Study design

Information about the study was performed in two steps. The staffs of the refugee centres were asked to participate in this observational study and detailed written information regarding the study design was provided. The staff of the local centre gave primary information to the residents. Those willing to participate were invited for a second, individual information about the details and procedure of the study given by the investigators in oral form during the site visit. Due to illiteracy, written informed consent was not feasible. Refugees who declined to participate were taken off the list provided by the centre staff. It was noted that the participants accepted the different swabs taken. Some accepted nasopharygeal but declined groin and/or rectal swabs. This was noted for each participant. Persons younger than 15 years were excluded from the study. If requested by the parents, we performed a clinical check and treated if for example scabies was present. However, no swabs were taken from these children. Persons 15 to 18 years were included, but all of them were young males that had travelled by themselves to Switzerland. This procedure was approved by the local ethic committee (EKNZ 2016–00005) and by the office of public health of the canton Solothurn (S1 File. approval ethic committee.pdf).

261 refugees participated in the study. The total number of refugees changed daily, but about 330 refugees were housed in the four centres. Participants were asked about skin problems, and if present, clinical evaluation was performed. For specific skin disease treatment was offered. In respect to travel time, some persons had spent years in another country before entering Switzerland, so we calculated travel time for these persons separately.

### Inclusion and exclusion criteria

20 participants declined groin or rectal swab sampling. They were excluded from the ESBL and carbapenemase- but still included in the MRSA screening.

Children younger than 15 years were not included, but were offered a clinical check if skin problems were present.

### Microbiological assessment

For detection of MRSA, swabs from throat, nostrils and groins were used. Swabs were cultured in Staphylococcus enrichment broth (AxonLab, Switzerland) and the following day subcultured on Columbia CNA with 5% sheep blood and ChromID^™^ MRSA smart (all bioMérieux, Geneva, Switzerland). Presumptive MRSA was confirmed by agglutination with Pastorex StaphPlus (BioRad, Switzerland) and Slidex^®^ MRSA (bioMérieux), respectively.

For detection of Enterobacteriaceae producing ESBL or carbapenemases, rectal swab and urine sampling was performed. Swabs and urine were directly cultured on MacConkey agar, ChromID^™^ ESBL, and ChromID^™^ Carba smart (all bioMérieux). Presumptive *Enterobacteriaceae* with ESBL production were identified by VitekMS (bioMérieux) and confirmed by double-disk synergy test (DDST) with cefepime and amoxicillin-clavulanic acid and by the combination disk test (CDT) with cefotaxime/ceftazidime +/- clavulanic acid (Becton Dickinson, Basle, Switzerland) according to EUCAST guidelines for detection of resistance mechanisms and specific resistances of clinical and/or epidemiological importance (Version 1.0; December 2013; page 14 ff).

For the screening of *Enterobacteriaceae producing carbapenemases*, all directly cultivated samples did not show any growth on ChromID^™^ Carba smart agar after two days of incubation.

Scabies was diagnosed clinically. In unclear situations, improvement after treatment with Ivermectin was estimated as being diagnostic.

### Whole genome sequencing

Due to a potential outbreak with MRSA, whole genome sequencing and analysis of genomic data was carried out as described previously [8]. Additionally, NGS analysis was carried out using Seqsphere+ (Ridom, Germany), the MRSA cgMLST scheme [9] and a maximum of 10 allelic differences was defined to be consistent with an transmission event. The data was analysed to determine the relatedness in phylogeny (regarding allelic differences) and to detect the PVL gene as an important virulence factor. All sequencing data is available from the Sequence Read Archive (http://www.ncbi.nlm.nih.gov/sra).

### Outbreak definition

In one centre, we saw a marked higher number of MRSA cases than in other centres. We defined an outbreak as significant higher proportion of MDR cases than expected that were geographically localized with evidence of transmission within a centre.

### Statistical analysis

Mean and standard deviations are indicated if not otherwise stated. The percentages of refugees screened positive for MRSA, ESBL and carbapenemases, and scabies were correlated with gender, the country of origin, the resident centre, and presence of skin disease. Categorical variables were analysed using a Chi-square test. All p-values are two sided with a 0.05 level of significance. Data was managed and analysed with the SPSS Statistics software (version 22.0; IBM, Chicago, IL, U.S.A.) and GraphPad Prism (version 4.0; GraphPad, La Jolla, CA, U.S.A.).

## Results

### Patient and travel characteristics

A total of 261 persons were screened in four centres. Centre A (47 persons) and centre D (55 persons) hosted single persons as well as families, while centre B (37 persons) and D (122 persons) hosted only men. Centre B and D were civil protection facilities located underground with up to 30 persons in one room. Centre A and C were normal buildings with smaller rooms and a maximum of six beds per room.

Although a total of 24 nationalities were present, most refugees originated from Afghanistan (92/261; 35.2%), Syria (39/261; 14.9%) and Eritrea (52/261; 19.9%). We classified them in five different regions: Middle East (Afghanistan, Iran, Iraq, Syria, Lebanon, Pakistan) East Africa (Eritrea, Ethiopia, Djibouti, Somalia, Sudan), Central/West Africa (Congo, Nigeria, Gambia, Ghana, Ivory Coast), Northern Africa (Egypt, Tunisia, Morocco), and Far East (China, Tibet, Sri Lanka, Bangladesh).

75% of the persons were male, mean age was 26.3 ± 8.3 years. Centre A hosted more persons from East Africa, whereas Centre B and D hosted more people from Middle East (Table 1). Mean stay in the centre before screening was 61.8 ± 46.7 days, without a significant difference between the centres (p = 0.20) (Table 1**)**. Overall, the refugees represent a largely heterogeneous group.

**Table 1:** Baseline charactreristics.

	Centre A	Centre B	Centre C	Centre D	total	p value
**Number**	47	55	37	122	261	
**Age (years)**	28.8±12.1	27.9±8.4	23.3±4.4	25.7±7.1	26.3±8.3	0.02
**Gender (% male)**	68	62	100	100	75	
**Region (n, %)**						<0.001
Middle East	16 (34)	34 (61.8)	20 (54)	82 (67.2)	152 (58.2)	
East Africa	20 (42.5)	13 (23.6)	12 (32.4)	27 (22.1)	72 (27.6)	
Northern Africa	4 (8.5)	0 (0)	0 (0)	4 (3.3)	8 (3.1)	
Central/West Africa	1 (2.1)	6 (11)	0 (0)	5 (4.1)	12 (4.6)	
Far East	6 (12.7)	2 (3.6)	5 (13.5)	4 (3.3)	17 (6.5)	
**Colonization rates**						
MRSA	4/47(8.5%)	5/55 (9.1%)	1/37(2.7%)	31/122(25.4%)	41/261(15.7%)	<0.001
ESBL E. coli	8/46 (17.4%)	13/54(24.1%)	9/37(24.3%)	27/104(26%)	57/241 (23.6%)	0.6
**Travel time (months)**	15.9 ± 24.8	5.7 ± 5.5	8.2 ± 47.8	10.8 ± 23.4	10.6 ± 21	0.44
**Travel time excluding persons with longer stay in third country (months)**	2.6 ± 1.5	4 ± 3.4	4 ± 3.4	2.6 ± 2.9	3.0 ± 2.9	0.22
**Duration of stay in centre (days)**	69.9 ± 79.7	63.8 ± 44	47.8 ± 37.0	62.5 ± 33.5	61.8 ± 46.7	0.2

Age, travel time and duration of stay in one particular centre are shown as mean +/- standard deviations

### Colonization rates with MRSA and ESBL/carbapenemase producers

**Fig 1:**
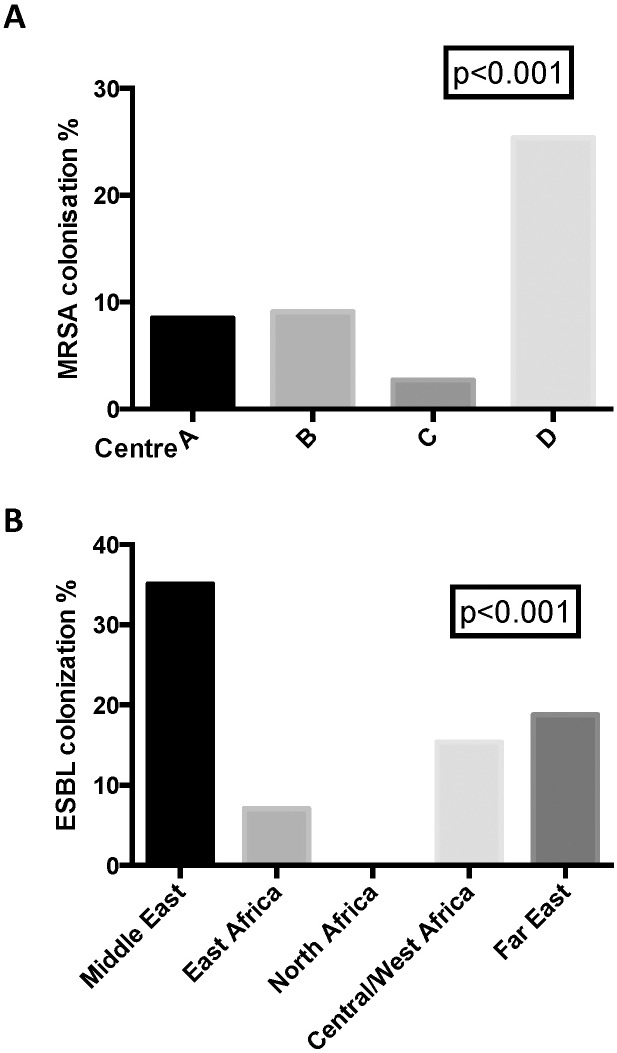
MRSA colonisation rates per centre and ESBL colonisation rate country of origin.

MRSA colonisation rate was 15.7% (41/261). We observed a significant difference between the centres (p<0.001; Fig 1), whereas no difference regarding the region of origin was found (p = 0.11, Table 2). One single refugee centre (centre D) showed a particular high colonization rate of 25.4%, suggesting an outbreak (see later). For detailed information concerning MLST sequence types see supplementary file.

**Table 2:** MRSA and ESBL prevalence according to regions.

MRSA	n	positiv	%	p-value
Middle East	151	18	11.9	0.11
North Africa	9	2	33.3	
East Africa	71	17	23.9	
Central/West Africa	13	2	15.4	
Far East	17	2	11.8	
**total**	261	41	16.1	
**ESBL**				
Middle East	134	47	35.1	<0.001
North Africa	9	0	0.0	
East Africa	70	5	7.1	
Central/West Africa	13	2	15.4	
Far East	16	3	18.8	
**total**	241	57	23.7	

30/261 persons (11.5%) suffered from scabies, with the highest rate in persons from Eritrea (23/52; 42.5%). Moreover, 17/261 persons had other non-scabies related skin affections such as wounds from injuries. Having skin affections was a risk factor for colonisation with MRSA (odds ratio 2.6, 95% CI 1.25–5.48, p = 0.01).

For ESBL, the overall rate of colonization was higher than for MRSA. A total of 57/241 (23.6%) were positive. In contrast to MRSA, no particular association with a specific refugee centre could be determined (p = 0.6). However, significantly more persons from Middle East were found to be positive compared to persons from other regions (p <0.001). In a post hoc analysis for ESBL colonisation rates, only the difference between Middle East and East Africa reached statistical significance (Odds ratio 7.02; p<0.001). However, the limited number of participants from the other regions was probably the factor for not reaching statistical significance. All ESBL strains were *E coli*, and no carbapenemase positive bacterial isolates were identified.

### Phylogenetic analysis of MRSA isolates

**Fig 2:**
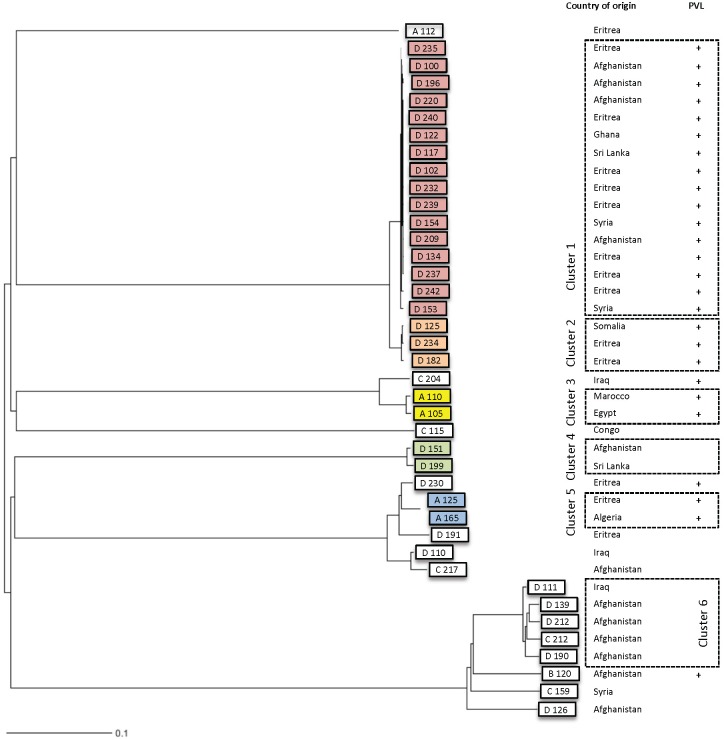
Phylogeny using whole genome sequencing data of MRSA isolates. Centres are marked as A-D. Panton valentin leukocidin (PVL) positive isolates are indicates with “+”.

Due to a potential outbreak in refugee centre D, we performed a whole genome sequencing (WGS) analysis of 40/42 available MRSA isolates. WGS phylogeny analysis of all MRSA strains is shown in Fig 2 using a neighbour joining tree. Six clusters with potential transmission events could be observed. One large cluster in refugee centre D showed 16 highly related isolates with less than ≤2 allele difference (cluster 1). Close to this cluster, we found a second smaller cluster with three more isolates (cluster 2, ≤3 allele difference). Very likely, these two clusters are connected, which was indicated due to 26 allelic differences between the two clusters (S1 Fig). Additionally, we identified three clusters with single transmission events, again less than ≤5 allelic differences apart (cluster 3–5). While isolates not found in those clusters differ by more than 250 alleles.

The countries of origin of the refugees within the clusters were scattered over all countries, no regional relatedness or similar travel route within the cluster could be identified. Moreover, the close relatedness (with less than 5 allelic differences) between the isolates of each cluster strongly indicates recent transmission events. One slightly more diverse cluster (cluster 6) showed about between 20 and 50 allelic differences between isolates and this suggests a much earlier transmission event (Fig 2 and S1 Fig).

When we accounted each outbreak and transmission event for one (initial) carrier, the estimated prevalence rate of MRSA was (20/261) 7.7%. In this calculation, the isolates of cluster 6 were counted as independent strains, as the diversity within the cluster suggested a transmission months ago.

### Virulence factors in MRSA isolates

We focused on the most dominating virulence factor, the Panton-Valentine leukocidin (PVL). Interestingly, we found that 26/40 (63%) of the samples were positive. This high rate could be largely explained due to the transmission of PVL-positive strains between the refugees (Fig 2**)**

### Antibiotic susceptibilities to non betalactame antibiotics (MRSA)

The susceptibilities were as following: clindamycin 39/39 (100%); erythromycin 38/39 (97.4%); TMP/SMP32/39 (82%); fusidic acid 18/39 (46.2%); tetracycline 10/39 (25.6%); quinolones 36/39 (92.3%), rifampin 39/93 (100%)

## Discussion

The colonisation rates of MDR bacteria is influenced by the regions and populations studied. Our study showed a MRSA carriage rate of 15.7%, which seems to be much higher compared to most European countries with rates of around 0.5–2% [10–12]. However, outside of Europe higher rates have been described. The nasal MRSA carriage rate range from 3.6%[13] (Taiwan janitors workers) to 32% and 25% in outpatients from Egypt and Saudi Arabia, respectively[14]. In our study, no statistically different MRSA carriage rates between different geographical regions were detected. We identified a larger outbreak in one of the Swiss refugees camps and a series of smaller transmission events. As a consequence, the “true” prevalence rate in the country of origin is not reliably assessable in such a cross-sectional study design. WGS analysis provided a high resolution to address this important caveat. When we accounted each outbreak for one (initial) carrier, the estimated prevalence rate of MRSA was 7.7%. We however think that the situation of refugees in the centres facilitates transmission, and outbreaks could also occur in other centres. The reason why the MRSA rate is much higher in the developing rather than in the western world is poorly understood, and data from origin countries of the refugees are scarce. However, our findings confirm some more recent work. In Ethiopia, the overall MRSA colonisation in health care workers was 12.7%[15]. In Eritrea, 9% of all *S*. *aureus* from healthy hospital staff was found to be resistant to methicillin[16]. For Syria and Afghanistan, no clear data about prevalence of MRSA exists, but case series with MRSA infections have been described [17,18]. However, the “true” prevalence rate might be over-estimated due to local outbreaks. For physicians treating patients from a refugee centre, local surveillance of refugee centres in regards of MDR bacteria might be very important. In our cross-sectional study, the MRSA colonization rate between refugee centres ranged from 2.4% to 25.4%. The high rate of PVL positive MRSA strains is interesting, and a recent comparison between Eritrean and Non-Eritrean patients with common skin infections found a higher percentage of PVL in MSSA of Eritrean patients as well [19].

The overall colonization rates of ESBL detected in refugees (23.7%) was much higher compared to recent published data in the Swiss population. In 2013, 5/170 (2.8%) healthy people showed a colonization with ESBL-producing Enterobacteriaceae prior to travelling from Switzerland to South Asia [20]. In a health care setting, higher numbers have been published. The Swiss antibiotic resistance surveillance program, Anresis, determined that 10.8% of *E*. *coli* isolated in 2015 were ESBL-producers [20]. Other studies from Switzerland and Spain describe similar rates of 5.8% in 2011 and 6.6% in 2006, respectively in the environment (food, animals)[21–23]. We could not determine differences in colonization rates between centres, but actually based on the country of origin. This provides some evidence that there was no local outbreak with ESBL *E*. *coli*.

No data is available from Eritrea regarding MDR *Enterobacteriaceae*. However, publications from Ethiopia and Afghanistan describe high rates of ESBL-producers with 87.4% and 70%, respectively[24,25]. In French military personnel returning from overseas (mainly Afghanistan), ESBL colonisation rate was 13.3%, and thus six times higher than reported in soldiers in suburbs of Paris[26]. We did not find carbapenemase producing bacteria in our cross-sectional screening. In Ethiopia 5/183 (2.7%) *Enterobacteriaceae* identified in urinary tract infection showed a Carbapenemase [24].

Our study has some limitations: Although this is the largest study investigating colonisation rates of MRSA, ESBL, and carbapenemases in refugees, it was limited to four centres of one geographical region of Switzerland. Travel history could not be exactly documented, and only persons newly arrived in Switzerland were included. As the dynamics of refugees arriving in Western countries is rapidly changing, our study provides just a snapshot. Unfortunately, the ESBL isolates were not available for WGS typing, so person to person transmission within a centre cannot definitively ruled out.

In summary, our data suggests, that for all refugees admitted to medical care facilities in Europe, screening and potentially contact isolation should be performed. A similar suggestion was made due to an observation in refugees admitted to a german university hospital with even higher rates of MDR gram negative bacteria in clinical specimen[27].The higher likelihood of MRSA and ESBL *E*. *coli* needs to be considered in the clinical evaluation of infectious diseases and covered by the choice of empirical treatment. A possible solution for faster information on presence of MDR pathogens in a refugee could be a central database for carriage of MDR bacteria or forwarding of the medical history to the subsequent refugee centre to reduce multiple screening. Until now, special focus of health care problems in migrants did not get much attention. Given the special situation, we think that “migrant medicine” has to become an important issue, and as these people often do not reflect epidemiology of classical European patients. Therefore more data, and especially longitudinal studies, are warranted to assess these important aspects impacting on public health.

## Supporting Information

S1 Fig Burst diagram based on core genome MLST. (TIFF)

S1 File Approval ethic committee. (PDF)

S1 Table Supple-MLST-ST. (DOCX)
