# Correction: A Cross-Sectional Study of Colonization Rates with Methicillin-Resistant *Staphylococcus aureus* (MRSA) and Extended-Spectrum Beta-Lactamase (ESBL) and Carbapenemase-Producing *Enterobacteriaceae* in Four Swiss Refugee Centres

**DOI:** 10.1371/journal.pone.0174911

**Published:** 2017-03-27

**Authors:** Rein Jan Piso, Roman Käch, Roxana Pop, Daniela Zillig, Urs Schibli, Stefano Bassetti, Dominik M. Meinel, Adrian Egli

The middle initial of the seventh author's name is omitted. The correct name is: Dominik M. Meinel.
