# Supplementary figures and images for: A Cross-Sectional Study of Colonization Rates with Methicillin-Resistant Staphylococcus aureus (MRSA) and Extended-Spectrum Beta-Lactamase (ESBL) and Carbapenemase-Producing Enterobacteriaceae in Four Swiss Refugee Centres
Source: PLoS One. 2017 Jan 13;12(1):e0170251. doi: 10.1371/journal.pone.0170251 (PMC5234815; doi:10.1371/journal.pone.0170251)

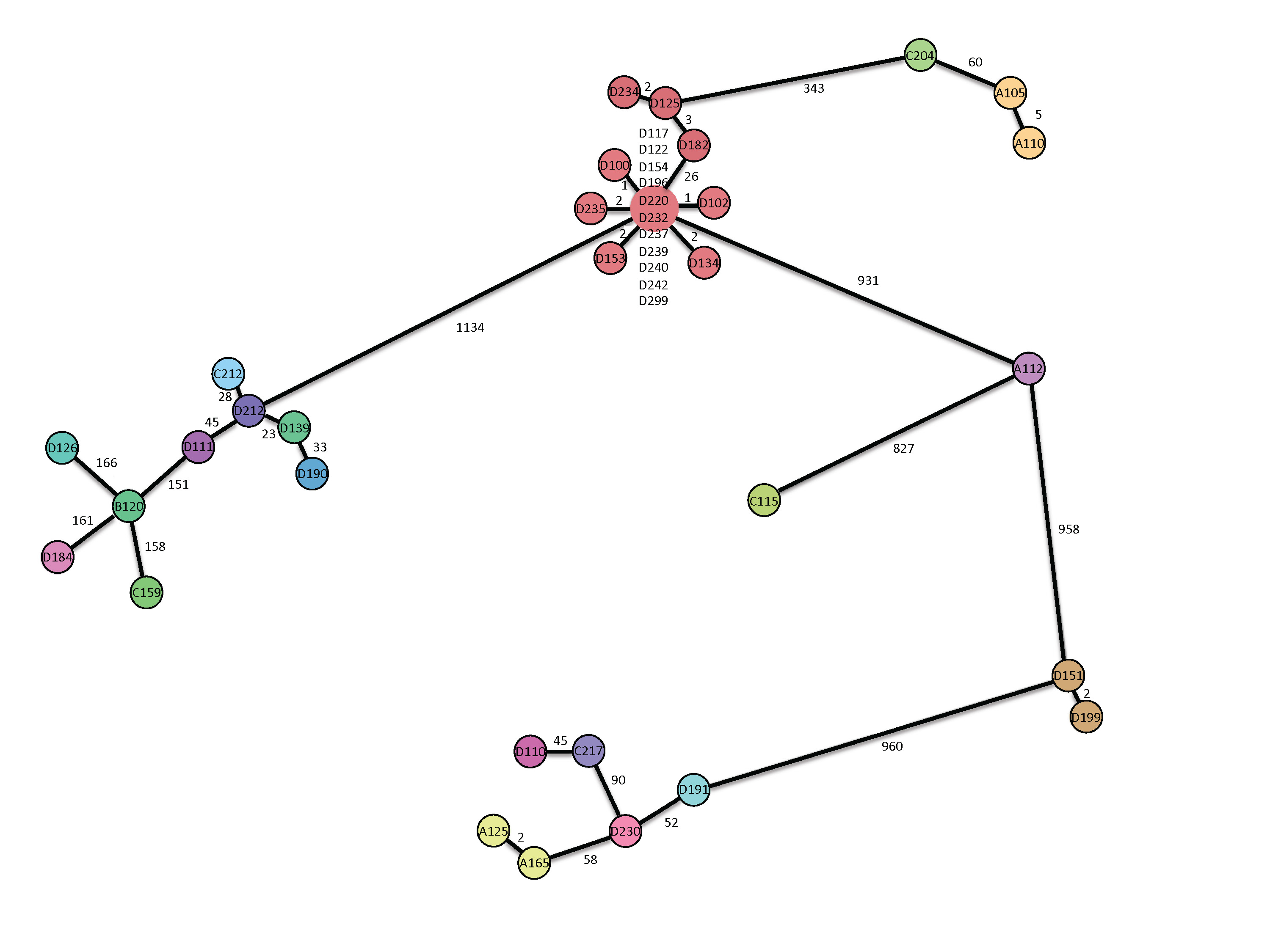

Supplement: S1 Fig — (TIFF) [file pone.0170251.s001.tiff]
