# Supplementary material for: A Cross-Sectional Study of Colonization Rates with Methicillin-Resistant Staphylococcus aureus (MRSA) and Extended-Spectrum Beta-Lactamase (ESBL) and Carbapenemase-Producing Enterobacteriaceae in Four Swiss Refugee Centres
Source: PLoS One. 2017 Jan 13;12(1):e0170251. doi: 10.1371/journal.pone.0170251 (PMC5234815; doi:10.1371/journal.pone.0170251)
Supplement: S1 File — (PDF) [file pone.0170251.s002.pdf]

Ethikkommission Nordwest- und Zentralschweiz EKNZ

Präsident
Prof. André P. Perruchoud
Vizepräsidenten
Prof. Gregor Schubiger
Dr. Marco Schärer

Herr
Dr. med. Rein Jan Piso
Kantonsspital Olten
Baslerstrasse 150
4600 Olten

Basel, 01. Februar 2016 / NJ

EKNZ 2016-00005:
Prevalence of MRSA and ESBL in Four Swiss Refugee Centres

Sehr geehrter Herr Dr. Piso

Besten Dank für Ihre Einreichung datiert vom 5. Januar 2016 samt Beilagen. Die Ethikkommission Nordwest- und Zentralschweiz hat die Dokumente zum oben genannten Projekt zur Kenntnis genommen und nimmt nun wie folgt Stellung:

Unsere Abklärungen haben ergeben, dass es sich um keine bewilligungspflichtige Studie im Sinne der kantonalen und eidgenössischen Gesetzgebung handelt, weil es eine Qualitätskontrolle betrifft. Aus diesem Grund kann die EKNZ keine förmliche Bewilligung ausstellen. Nach Überprüfung der Anfrage kann die EKNZ jedoch feststellen, dass die Durchführung dieser Studie aus ethischer Sicht unbedenklich ist (vgl. Art. 51 Abs. 2 Humanforschungsgesetz).

Ich hoffe, Ihnen mit diesen Angaben zu dienen und verbleibe

mit freundlichen Grüssen

i.V. [illegible]

Prof. A. P. Perruchoud
Präsident der Ethikkommission
Nordwest- und Zentralschweiz / EKNZ
