# Supplementary material for: A Cross-Sectional Study of Colonization Rates with Methicillin-Resistant Staphylococcus aureus (MRSA) and Extended-Spectrum Beta-Lactamase (ESBL) and Carbapenemase-Producing Enterobacteriaceae in Four Swiss Refugee Centres
Source: PLoS One. 2017 Jan 13;12(1):e0170251. doi: 10.1371/journal.pone.0170251 (PMC5234815; doi:10.1371/journal.pone.0170251)
Supplement: S1 Table — (DOCX) [file pone.0170251.s003.docx]

S1 Table: MLST sequence types of the MRSA isolates according to the pubMLST.org database.

| Sample ID | ST | Profile | arcc | aroe | glpf | gmk | pta | tpi | yqil |
| --- | --- | --- | --- | --- | --- | --- | --- | --- | --- |
| D240 | 1 | 1, 1, 1, 1, 1, 1, 1 | 1 | 1 | 1 | 1 | 1 | 1 | 1 |
| D239 | 1 | 1, 1, 1, 1, 1, 1, 1 | 1 | 1 | 1 | 1 | 1 | 1 | 1 |
| D117 | 1 | 1, 1, 1, 1, 1, 1, 1 | 1 | 1 | 1 | 1 | 1 | 1 | 1 |
| D134 | 1 | 1, 1, 1, 1, 1, 1, 1 | 1 | 1 | 1 | 1 | 1 | 1 | 1 |
| D196 | 1 | 1, 1, 1, 1, 1, 1, 1 | 1 | 1 | 1 | 1 | 1 | 1 | 1 |
| D232 | 1 | 1, 1, 1, 1, 1, 1, 1 | 1 | 1 | 1 | 1 | 1 | 1 | 1 |
| D234 | 1 | 1, 1, 1, 1, 1, 1, 1 | 1 | 1 | 1 | 1 | 1 | 1 | 1 |
| D220 | 1 | 1, 1, 1, 1, 1, 1, 1 | 1 | 1 | 1 | 1 | 1 | 1 | 1 |
| D154 | 1 | 1, 1, 1, 1, 1, 1, 1 | 1 | 1 | 1 | 1 | 1 | 1 | 1 |
| D139 | 22 | 7, 6, 1, 5, 8, 8, 6 | 7 | 6 | 1 | 5 | 8 | 8 | 6 |
| D190 | 22 | 7, 6, 1, 5, 8, 8, 6 | 7 | 6 | 1 | 5 | 8 | 8 | 6 |
| D122 | 1 | 1, 1, 1, 1, 1, 1, 1 | 1 | 1 | 1 | 1 | 1 | 1 | 1 |
| D184 | 737 | 7, 6, 1, 70, 8, 8, 6 | 7 | 6 | 1 | 70 | 8 | 8 | 6 |
| C115 | 8 | 3, 3, 1, 1, 4, 4, 3 | 3 | 3 | 1 | 1 | 4 | 4 | 3 |
| D125 | 1 | 1, 1, 1, 1, 1, 1, 1 | 1 | 1 | 1 | 1 | 1 | 1 | 1 |
| A110 | 80 | 1, 3, 1, 14, 11, 51, 10 | 1 | 3 | 1 | 14 | 11 | 51 | 10 |
| D212 | 22 | 7, 6, 1, 5, 8, 8, 6 | 7 | 6 | 1 | 5 | 8 | 8 | 6 |
| C217 | 859 | 79, 1, 14, 23, 12, 4, 31 | 79 | 1 | 14 | 23 | 12 | 4 | 31 |
| C212 | 22 | 7, 6, 1, 5, 8, 8, 6 | 7 | 6 | 1 | 5 | 8 | 8 | 6 |
| C159 | 22 | 7, 6, 1, 5, 8, 8, 6 | 7 | 6 | 1 | 5 | 8 | 8 | 6 |
| A112 | 15 | 13, 13, 1, 1, 12, 11, 13 | 13 | 13 | 1 | 1 | 12 | 11 | 13 |
| D111 | **US1** | 7, ?, 1, 5, 8, 8, 6 | 7 | **UA1** | 1 | 5 | 8 | 8 | 6 |
| D110 | 859 | 79, 1, 14, 23, 12, 4, 31 | 79 | 1 | 14 | 23 | 12 | 4 | 31 |
| D230 | 88 | 22, 1, 14, 23, 12, 4, 31 | 22 | 1 | 14 | 23 | 12 | 4 | 31 |
| D199 | 3841 | 12, 4, 1, 4, 12, 403, 3 | 12 | 4 | 1 | 4 | 12 | 403 | 3 |
| D100 | 1 | 1, ?, 1, 1, 1, 1, 1 | 1 | 1 | 1 | 1 | 1 | 1 | 1 |
| A125 | **US2** | ?, 1, 14, 23, 12, 4, 31 | **UA2** | 1 | 14 | 23 | 12 | 4 | 31 |
| D235 | 1 | 1, 1, 1, 1, 1, 1, 1 | 1 | 1 | 1 | 1 | 1 | 1 | 1 |
| A165 | **US2** | ?, 1, 14, 23, 12, 4, 31 | **UA2** | 1 | 14 | 23 | 12 | 4 | 31 |
| D182 | 1 | 1, 1, 1, 1, 1, 1, 1 | 1 | 1 | 1 | 1 | 1 | 1 | 1 |
| D151 | 3841 | 12, 4, 1, 4, 12, 403, 3 | 12 | 4 | 1 | 4 | 12 | 403 | 3 |
| C204 | 80 | 1, 3, 1, 14, 11, 51, 10 | 1 | 3 | 1 | 14 | 11 | 51 | 10 |
| D237 | 1 | 1, 1, 1, 1, 1, 1, 1 | 1 | 1 | 1 | 1 | 1 | 1 | 1 |
| D153 | 1 | 1, 1, 1, 1, 1, 1, 1 | 1 | 1 | 1 | 1 | 1 | 1 | 1 |
| D126 | 22 | 7, 6, 1, 5, 8, 8, 6 | 7 | 6 | 1 | 5 | 8 | 8 | 6 |
| A105 | 80 | 1, 3, 1, 14, 11, 51, 10 | 1 | 3 | 1 | 14 | 11 | 51 | 10 |
| D242 | 1 | 1, 1, 1, 1, 1, 1, 1 | 1 | 1 | 1 | 1 | 1 | 1 | 1 |
| D191 | 88 | 22, 1, 14, 23, 12, 4, 31 | 22 | 1 | 14 | 23 | 12 | 4 | 31 |
| D209 | 1 | 1, 1, 1, 1, 1, 1, 1 | 1 | 1 | 1 | 1 | 1 | 1 | 1 |
| D102 | 1 | 1, 1, 1, 1, 1, 1, 1 | 1 | 1 | 1 | 1 | 1 | 1 | 1 |
| B120 | 22 | 7, 6, 1, 5, 8, 8, 6 | 7 | 6 | 1 | 5 | 8 | 8 | 6 |

ST: sequence type

UA1 and UA2: previously unknown allele. Submitted to pubMLST.org

US1 and US2: previously unknown sequence type. Submitted to pubMLST.org
